# Identification and validation of an immune cell infiltrating score predicting survival in patients with lung adenocarcinoma

**DOI:** 10.1186/s12967-019-1964-6

**Published:** 2019-07-08

**Authors:** Xiaodong Yang, Yu Shi, Ming Li, Tao Lu, Junjie Xi, Zongwu Lin, Wei Jiang, Weigang Guo, Cheng Zhan, Qun Wang

**Affiliations:** Department of Thoracic Surgery, Zhongshan Hospital, Fudan University, No. 180, Fenglin Road, Shanghai, 200032 China

**Keywords:** Lung adenocarcinoma, Score, Prognosis, Immune infiltration

## Abstract

**Background:**

Immune infiltration may predict survival and have clinical significance in lung cancer. However, immune signatures derived from immune profiling based on bulk tumor transcriptomes have not been systematically established in lung adenocarcinoma. We aimed to construct an immune cell infiltrating score, using a new algorithm for evaluating immune infiltration, to improve the prognostic model of lung adenocarcinoma.

**Methods:**

Public datasets of lung adenocarcinoma from the Gene Expression Omnibus and The Cancer Genome Atlas were adopted as the training and validation cohorts. Fractions of different immune cell subtypes in each sample were estimated using the CIBERSORT algorithm. The immune infiltrating score was further developed by a least absolute shrinkage and selection operator regression model. The prognostic value and clinical relationship of the model was then further explored.

**Results:**

An immune infiltrating score model was established on the basis of the immune cells in the training cohort. A high score was associated with significantly worse survival in patients with lung adenocarcinoma (P < 0.001). The prognostic value of the score was confirmed in the validation cohort. The immune infiltrating score could improve the accuracy of predictions of survival when combined with the staging system. Furthermore, the score was potentially associated with patient smoking status and histologic subtype of lung adenocarcinoma. Its possible association with the efficacy of adjuvant chemotherapy was not statistically significant.

**Conclusion:**

The immune cell infiltrating score has prognostic significance in predicting overall survival in patients with lung adenocarcinoma.

**Electronic supplementary material:**

The online version of this article (10.1186/s12967-019-1964-6) contains supplementary material, which is available to authorized users.

## Background

Lung cancer remains the leading cause of cancer incidence and death worldwide in 2018, when it was estimated to comprise 2.1 million new cases and cause 1.8 million deaths [1]. Among all patients with lung cancer, lung adenocarcinoma accounts for the largest proportion, and its incidence continues to increase [2]. The literature has shown that phenotypes of cancers are dependent on the tumor microenvironment, especially tumor-infiltrating immune cells [3, 4]. The immune response is characterized by many types of cells, and differences in their potential prognostic value depend on the cancer type [5]. Computational approaches were adopted to characterize immune-interactions in lung adenocarcinoma, mainly focused on B cell and CD8+ T cell [6]. Meta-analysis also showed that CD8+, CD3+ and FOXP3+ T cells infiltration had significantly prognostic value in lung adenocarcinoma [7]. Furthermore, the prognostic significance of integrated immune infiltration in some cancers has been validated, and this has been proposed to supplement the tumor, node, and metastasis (TNM) staging system for predicting patient survival [8–13]. However, very few studies reported integrated immune infiltration in lung adenocarcinoma.

Most previous studies have adopted an immunohistochemistry method to evaluate the landscape of tumor-infiltrating immune cell subtypes. However, a bioinformatics tool, known as the CIBERSORT algorithm, has been previously developed for computational enumeration of immune cell subtypes. The landscape and proportions of tumor-infiltrating immune cells are evaluated on the basis of the changes in expression of immune-relative and other genes in one sample. This meta-gene approach has been well designed and validated in previous studies [9, 14–16].

In the present study, CIBERSORT was used to estimate the fraction of immune cell types based on different lung adenocarcinoma gene expression profiles. Least absolute shrinkage and selection operator (LASSO) Cox regression analysis was utilized to establish an immune cell infiltrating score model in both training and validation cohorts. We believe that the immune infiltrating score could assist in predicting survival, along with clinical factors, in lung adenocarcinoma.

## Methods

### Study cohort and data processing

We adopted the public datasets from the Gene Expression Omnibus (GEO) (https://www.ncbi.nlm.nih.gov/geo/) as the training cohort. Four GEO datasets (GSE30219, GSE37745, GSE50081 and GSE68465, the latter also known as the Director’s Challenge Consortium) were included in this study. All of the four datasets were derived from the genechips of Affymetrix^®^ (Santa Clara, California, USA). GSE30219, GSE37745 and GSE50081 were based on the GPL570 genechip, while GSE68465 were from the GPL96 microarray. Then, raw data and genechip files were downloaded directly. A total of 85, 106, 127 and 443 samples of lung adenocarcinoma from GSE30219, GSE37745, GSE50081 and GSE68465 datasets were enrolled. We used a robust multichip average method via RMAExpress for background adjustment, quantile normalization and summary to process the gene profiles [17–19].

Level 3 RNA sequence data from lung adenocarcinoma samples were also downloaded from The Cancer Genome Atlas (TCGA) before June 5, 2018 (https://portal.gdc.cancer.gov/). A total of 594 samples were obtained, comprising 535 adenocarcinoma and 59 normal lung samples. Baseline clinicopathological factors and treatment information were also acquired from TCGA. Primary lung adenocarcinoma samples with complete follow-up and baseline information (age, sex and pathological stage) were included as the validation cohort in this study. Due to the original records of all included datasets, the seventh edition of the TNM staging method was utilized in our study. Information regarding histologic subtypes of lung adenocarcinoma was intergraded with records from the University of California Santa Cruz Xena database (http://xena.ucsc.edu/) and previous literature [20–22]. According to previous studies, tumors were classified into three subgroups (low-, intermediate- and high-grade adenocarcinoma) regarding the survival data for each predominant lung adenocarcinoma subtype [23, 24].

### Estimation of tumor-infiltrating immune cell types

The CIBERSORT method was adopted to quantify the proportions of the immune cell in both training and validation cohorts [25, 26]. Common methods, like flow cytometry and immunohistochemistry, rely on few phenotype markers and tissue disaggregation prior to cytometry, may cause damaged or lost cells, altering analyses [26]. The CIBERSORT was developed to accurately evaluate the relative levels of the 22 immune cell phenotypes, especially closely related types, using signature gens within complex expression mixtures [26]. Such mixtures could derive from both patients’ solid tissues and blood profiled by array or RNA-sequencing [26]. The 22 immune cells are mainly composed of B cells, T cells, macrophages, dendritic cells, plasma cells, natural killer cells and mast cells. To process data by the CIBERSORT, the targeted gene profile was uploaded to its website (https://cibersort.stanford.edu). Then, a heat-map table with levels of 22 immune cell subtypes returned as the final results. As previously described, we set the threshold *P*-value < 0.05 and excluded samples without immune infiltration in training (5 patients) and validation (78 patients) cohorts [25]. The optimal cut-off values of the proportions of different immune cells in the training cohort were calculated on the basis of the prognostic significance using X-Tile software [27]. Each immune cell fraction level of samples from both cohorts was then divided into two groups based on the cut-off point. The immune cell fraction level divided by the cut-off value was valued as 0 or 1 in the subsequent scoring formula.

### Statistical analysis

All statistical analyses in this study were performed using R version 3.5.0 (R Foundation for Statistical Computing, Vienna, Austria) and IBM SPSS Statistics 22.0 (IBM, Inc., Armonk, NY, USA). We used the LASSO Cox regression model to select the ideal prognostic model among the estimated immune cell subtypes using the glmnet package in R [28]. The optimal model parameter λ and corresponding coefficients were determined by tenfold cross validations. We chose λ via 1 − SE (standard error) criteria as previously recommended [8, 9]. Based on the final immune infiltrating score model and corresponding coefficients, a formula for the score was constructed using the training cohort. The sensitivity and specificity of the prognosis prediction of the immune cell infiltrating score were evaluated by a time-dependent receiver operating characteristic (ROC) curve in R software. The nearest neighbor estimation method in the ROC curve was adopted with cut-off times of 1, 3 and 5 years. Survival curves were estimated using the Kaplan–Meier method and the log-rank test was utilized for comparing survival curves. Univariate and multivariate Cox proportional hazard models were adopted to identify all significant prognostic factors. The prognostic value of the included variables was evaluated by Harrell’s concordance index (c-index) [29]. The score stratified by subgroups were compared using the one-way ANOVA method, if normal distribution and homogeneity of variance were followed. Correlation analysis was also conducted to explore the potential relationship between the score and the expression of immune checkpoint regulators. In this study, a two-tailed *P*-value of < 0.05 was considered statistically significant.

## Results

### Construction of the immune cell infiltrating score model

**Fig. 1:**
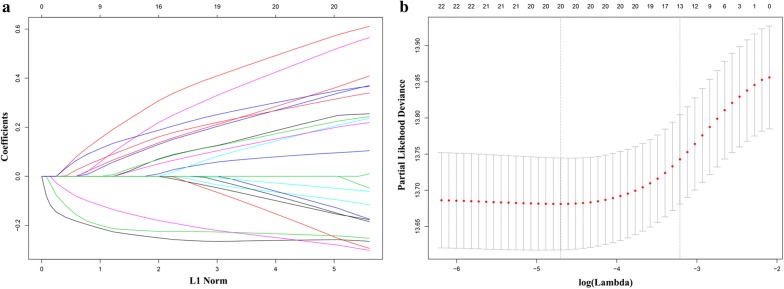
**a** Coefficient profiles of immune cell types in the LASSO Cox regression model. **b** Tenfold cross-validation for turning parameter selection in the LASSO Cox regression model. λ is the turning parameter. The partial likelihood deviance is plotted in log(λ), in which vertical lines are shown at the optimal values by minimum criteria and 1 − SE criteria

**Fig. 2:**
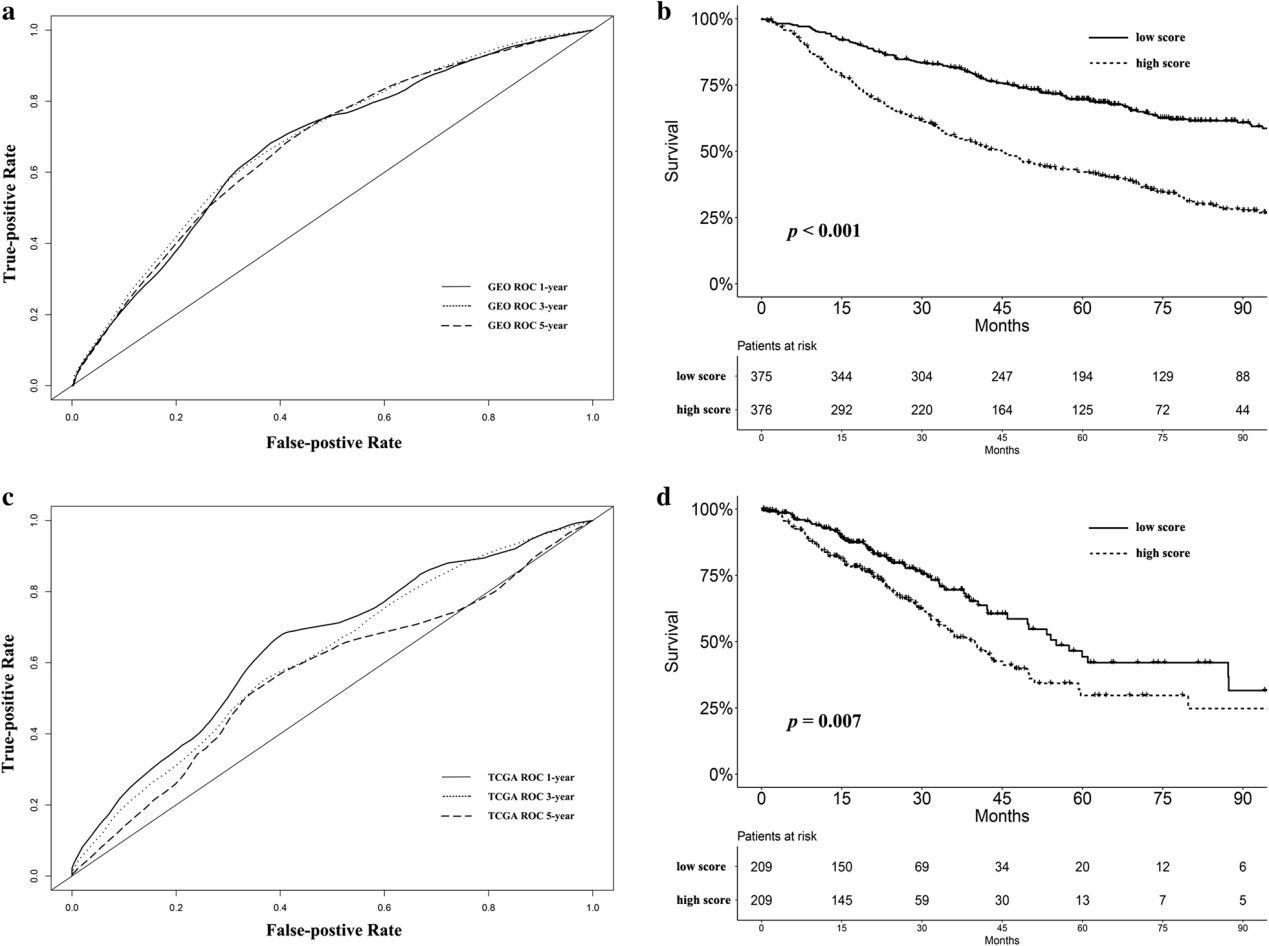
**a** The immune cell infiltrating score evaluated by time-dependent ROC curves at 1, 3 and 5 years in the training cohort. The area under the curve at each time point was 0.674, 0.684 and 0.675, respectively. **b** Survival analysis stratified by immune cell infiltrating score in the training cohort (*P *< 0.001). **c** The immune cell infiltrating score evaluated by time-dependent ROC curves at 1, 3 and 5 years in the validation cohort. The area under the curve at each time point was 0.650, 0.616 and 0.567, respectively. **d** Survival analysis stratified by immune cell infiltrating score in the validation cohort (*P *= 0.013)

After the initial selection process, 751 and 418 patients were enrolled as the training and validation cohorts, respectively. The characteristics of the patients are listed in Table 1 and Additional file 1: Table S1. Optimal cut-off values for the fraction of each immune cell type in the training cohort were generated as described in “Methods” section (Additional file 2: Table S2). LASSO Cox regression analysis was performed, based on the fraction of immune cells in the training cohort (Fig. 1a, b). Finally, the formula of the immune cell infiltrating score for lung adenocarcinoma in the training cohort was built (Additional file 3). The prognostic accuracy of the scoring model was evaluated using time-dependent ROC analysis in the training cohort. The predictive time points were set at 1, 3 and 5 years, respectively (Fig. 2a). The area under the ROC curve for the immune infiltrating score at each time point was 0.674, 0.684 and 0.675, respectively.

**Table 1:** Baseline characteristics of patients with lung adenocarcinoma in the training cohort

Variable	Baseline characteristics (n = 751)
Affymetrix^®^ platform	
HG-U133 Plus 2.0 (GPL570)	313 (41.7)
HG-U133A (GPL96)	438 (58.3)
Age	64.6 ± 10.0
Sex	
Male	394 (52.5)
Female	357 (47.5)
Tumor stage	
Stage I	515 (68.6)
Stage II–III	232 (30.9)
Stage IV	4 (0.5)
Smoking status	
Current smoker	67 (8.9)
Ex-smoker	322 (42.9)
Non-smoker	71 (9.5)
Unknown	291 (38.7)
Adjuvant chemotherapy	
Yes	102 (13.6)
No	377 (50.2)
Unknown	272 (36.2)

Continuous data (age) was presented as mean ± standard deviation and categorical data as number (proportion of that the subgroup accounted for the whole group)

Survival analyses were performed stratified by the score, in the training cohort (Fig. 2b). Patients were separated into low- and high-score groups by the median score of the cohort. We observed that patients with a high score had significantly worse survival than those with a low score (*P *< 0.001, Fig. 2b). A high score was also associated with significantly shorter progression-free survival (*P *< 0.001, Additional file 4: Figure S1).

As a continuous variable, the immune cell infiltrating score was found to have significantly prognostic value using univariate Cox regression analysis (*P* < 0.001, Table 2). Multivariate analysis confirmed that the immune infiltrating score was an independent prognostic factor in the training cohort, along with the TNM staging system (*P* < 0.001, Table 2). According to the corresponding c-index, the combination of the scoring model and TNM stage showed a significantly better predictive value than the TNM stage alone in this cohort (*P* < 0.001, Table 3).

**Table 2:** Results of univariate and multivariate Cox regression of overall survival in the training cohort

Cohort	Training cohort					
Variable	Univariate			Multivariate		
	HR	95% CI	*P*-value	HR	95% CI	*P*-value
Immune infiltrating score	6.459	4.409–9.462	< 0.001	5.322	3.574–7.926	< 0.001
Age	1.025	1.014–1.035	< 0.001	1.024	1.014–1.035	< 0.001
Sex			0.031			0.079
Female	Reference			Reference		
Male	1.245	1.021–1.518	0.031	1.196	0.979–1.460	0.079
TNM stage			< 0.001			< 0.001
Stage I	Reference			Reference		
Stage II–III	2.682	2.195–3.278	< 0.001	2.443	1.996–2.990	< 0.001
Stage IV	2.256	0.721–7.053	0.162	1.835	0.586–5.740	0.297

*HR* hazard ratio, *CI* confidence interval

**Table 3:** Comparison of the accuracy of survival prediction between the TNM stage with and without the immune infiltrating score

Cohort	TNM stage		Immune infiltrating score + TNM stage		*P*-value
	c-index	95% CI	c-index	95% CI	
Training cohort	0.619	0.594–0.643	0.686	0.659–0.714	< 0.001
Validation cohort	0.661	0.621–0.700	0.695	0.650–0.740	< 0.001

*CI* confidence interval

### Validation of the immune cell infiltrating score model

We adopted a validation group based on the dataset from TCGA to evaluate the prognostic value of the proposed scoring model. The same formula for the immune cell infiltrating score and optimal cut-off point for each immune cells were applied to the validation group. Likewise, ROC analysis was adopted to assess the prognostic value of the scoring model. The area under the curve was 0.650, 0.616 and 0.567 at the predictive time of 1, 3 and 5 years, respectively (Fig. 2c). In the validation group, a high score was associated with significantly worse prognosis (*P *= 0.013, Fig. 2d).

Consistent with the previous findings, the score was an independent prognostic factor in the validation cohort, based on the univariate and multivariate Cox regression model (*P* = 0.001 and 0.002, Additional file 5: Table S3). Furthermore, the combination of the scoring model and TNM stage improved the prognostic model of lung adenocarcinoma in this cohort (*P* < 0.001, Table 3).

### Clinical significance and bioinformatics analyses based on the immune cell infiltrating score

**Fig. 3:**
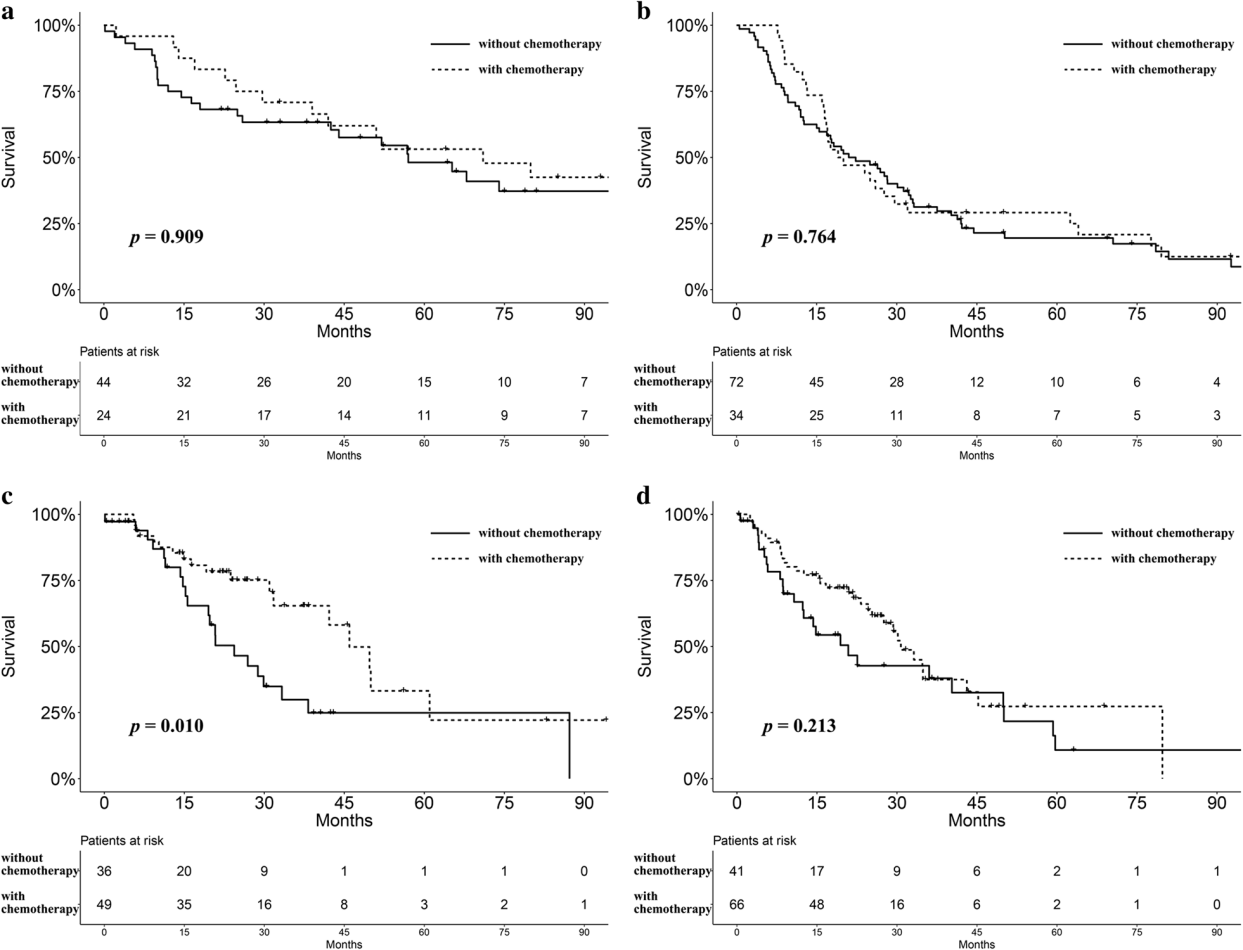
**a** Survival analysis for patients (stage II–IV) with low scores stratified by the receipt of adjuvant chemotherapy in the training cohort (*P* = 0.909). **b** Survival analysis for patients (stage II–IV) with high scores stratified by the receipt of adjuvant chemotherapy in the training cohort (*P* = 0.764). **c** Survival analysis for patients (stage II–IV) with low scores stratified by the receipt of adjuvant chemotherapy in the validation cohort (*P* = 0.010). **d** Survival analysis for patients (stage II–IV) with high scores stratified by the receipt of adjuvant chemotherapy in the validation cohort (*P* = 0.213)

In the training group, data regarding adjuvant chemotherapy were documented only in the GSE68465 and GSE37745 datasets. Patients were divided into low and high score groups based on the score median value. Given the differences in staging methods and initial study design, patients with stage I disease were excluded from this analysis. The survival advantage of the low-score group was observed in patients with or without adjuvant chemotherapy (*P* = 0.004 and 0.002, Additional file 6: Figure S2A, B). Moreover, there was a trend for better prognosis in patients with a low score who received adjuvant chemotherapy, although the difference was not statistically significant (*P* = 0.909, Fig. 3a). No significant prognostic difference regarding adjuvant chemotherapy was observed in high score group (*P* = 0.764, Fig. 3b). We also extracted data on adjuvant chemotherapy from the validation group. There was a trend for better survival in low score group who received chemotherapy (*P* = 0.084, Additional file 6: Figure S2C). No difference was found in patients without chemotherapy (*P* = 0.761, Additional file 6: Figure S2D). Furthermore, patients with a low score may benefit from adjuvant chemotherapy (*P* = 0.010, Fig. 3c), while no difference was observed in high-score group (*P* = 0.213, Fig. 3d).

**Fig. 4:**
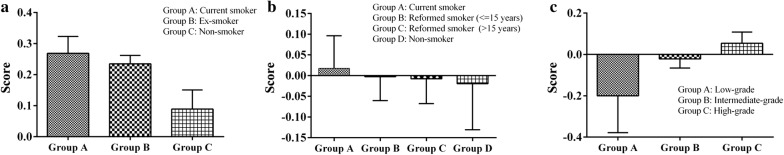
**a** Histograms of the immune cell infiltrating score stratified by the patient smoking status in the training cohort (*P* < 0.001). **b** Histograms of the immune cell infiltrating score stratified by the patient smoking status in the validation cohort (*P* = 0.932). **c** Histograms of the immune cell infiltrating score stratified by the histologic subtype of lung adenocarcinoma in the validation cohort (*P* = 0.003). High-grade: micropapillary or solid predominant lung adenocarcinoma; intermediate-grade: acinar or papillary predominant lung adenocarcinoma; low-grade: lepidic predominant lung adenocarcinoma

Smoking status was included in two datasets (GSE50081 and GSE68465) for the training cohort. Patients with unknown smoking status were excluded. We observed that patients who are current smokers had significantly higher score than those who do not smoke (*P* < 0.001, Fig. 4a). In the validation cohort, patients who are current or reformed smokers (< 15 years) had higher scores than non-smokers or reformed smokers (> 15 years), although the difference was not statistically significant (*P* = 0.932, Fig. 4b).

Furthermore, we explored potential relationships between the immune cell infiltrating score and histologic subtypes of lung adenocarcinoma. In the validation cohort, 186 patients with information on histologic subtypes were included. We found that high-grade adenocarcinoma subtypes were associated with significantly higher score than low-grade tumors (*P* = 0.003, micropapillary/solid vs acinar/papillary vs lepidic predominant lung adenocarcinoma, Fig. 4c).

Owing to the difference of the microarray platforms used for the training cohort, correlation analyses between the immune infiltrating score and the expression of immune checkpoint regulators or inflammatory mediators were performed in the validation cohort. We found that there was a significant positive correlation between the score and some of the regulators and mediators, including *PD*-*L1* (*P* = 0.002), *IDO1* (*P* = 0.003) and *LAG3* (*P* = 0.017), *IFNB1* (*P* = 0.002), *IL*-*1A* (*P* = 0.005), *TNFA* (*P* = 0.010) and *IL*-*6* (*P* = 0.007) (Additional file 7: Table S4).

## Discussion

In this study, we adopted the CIBERSORT method to calculate the fraction of 22 immune cell in lung adenocarcinoma. This newly developed algorithm can work accurately for bulk tumor samples profiled by microarray or RNA-Sequencing. And unlike flow or mass cytometry-based methods, it is applicable to archived RNA and cellular specimens [26]. Previous studies have validated the efficacy of the CIBERSORT method [14–16]. On the basis of the estimated fractions of signature immune cells, LASSO regression was utilized to construct an immune cell infiltrating score model, of which the predictive accuracy has been demonstrated previously [14, 28, 30, 31]. In both cohorts, the prognostic value of the scoring model was confirmed. A lower score was associated with significantly better prognosis, which may be due to more immunity-activating lymphocyte infiltrations. Further analyses also implied that the score could significantly improve the accuracy of survival prediction when combined with the TNM staging system. Significant correlation between the score and the expression of common immune checkpoint regulators or inflammatory mediators was also observed, including *PD*-*L1*, *IDO1* and *LAG3*, which further confirmed its potential values. Previous studies also indicated that the expression of *PD*-*L1* and *LAG*-*3* was related with early recurrence and poor prognosis [32, 33]. In a word, we hope that our work will provide new insights into the construction of staging or prognostic models in lung adenocarcinoma.

The role of different tumor-infiltrating lymphocytes in lung adenocarcinoma has been explored separately [7, 34]. Kinoshita et al. [35] found that CD8+ T-cells accumulation was identified as a negative prognostic factor in lung adenocarcinoma, but not in lung squamous carcinoma. Comprehensive immune profiling of lung adenocarcinoma revealed plasma cell infiltration was related to worse prognosis [36]. Mast cell exosomes were also reported to promote lung adenocarcinoma cell proliferation [37]. The function of mast cells against or in favor of the cancers still requires investigations [38, 39]. Previous studies also observed that the prognostic implication differs according to histological types and smoking status [35, 40]. The potential role of tumor-infiltrating lymphocytes may require exploration comprehensively according to immune microenvironment. The functionality of CD8+ tumor-infiltrating lymphocytes was reported to be affected by competition between antitumor and exhaustion programs [41]. Choi and Na investigated the relationship between the immune landscape and tumor glucose metabolism in lung adenocarcinoma [42]. Varn et al. adopted computational approaches to characterize tumor-immune interactions, mainly focused on six immune cell subtypes [6]. In our study, we adopted the CIBERSORT method with 22 cell phenotypes in lung adenocarcinoma like the study of Gentles et al. [5]. The final formula of the immune cell infiltrating score was composed of 13 types of immune cells using LASSO regression, which may better characterize the potential internal interactions and microenvironment.

Several studies have focused on the relationships between tumor-infiltrating lymphocytes and the efficacy of adjuvant chemotherapy [8, 43–45]. In this study, the potential associations with chemotherapy were not all statistically significant. The weak effect may be due to small sample size in this part of analyses and the difficulty in finding the optimal cut-off value for the score. Previous studies showed that the chemotherapy sensitivity may depend on the lymphocytes infiltrating the tumor [8, 9]. One potential mechanism is that the interferon secreted by lymphocytes could sensitize cells to chemotherapy [46]. The innate and adaptive immune responses may also be activated by immunogenic tumor cell death [47]. More investigations may be required for the underlying mechanisms [48]. Our study also indicated that the score was significantly associated with patient smoking status and histologic subtype, which may help us to better characterize patient subgroups and choose the optimal treatment.

The strength of this study is the large cohort derived from several institutions, which outnumbered than most similar researches of lung adenocarcinoma. We adopted a newly developed CIBERSORT method to estimate the level of tumor-infiltrating lymphocytes, which outperformed other methods (like immunohistochemistry) regarding noise, unknown mixture content and closely related cell types. There are also some limitations that should be noted. First, the public datasets in this study were based on different genechips, but we adopted the RMAExpress method which designed for Affymetrix^®^ microarrays and merged the raw data. All the datasets were collected with different study purposes, which may lead to heterogeneities in baseline features and therapies. Also, only retrospective analyses were made. Future prospective studies will be needed to confirm the findings. Second, some clinicopathologic factors were missing or incomplete, especially histological subtype, smoking status and adjuvant chemotherapy. Further investigations should collect more clinical factors and endpoints, along with possible alternative methods for testing and validation, which may provide more evidence for lung adenocarcinoma [13]. In addition, optimal cut-off value and practical approaches are essential for future clinical application.

## Conclusion

An immune cell infiltrating score model was established based on the immune cells in lung adenocarcinoma. A high score was associated with significantly worse survival. The scoring model could improve the accuracy of predictions of survival when combined with the staging system. It was also shown that the score was closely associated with the smoking status, histologic subtype and the expression of some immune checkpoint regulators or inflammatory mediators. This study may provide new insights into the construction of staging or prognostic models in lung adenocarcinoma. The close associations between the immune infiltrating score and clinical factors may help us to better identify patient subgroups and choose the optimal treatment for patients with lung adenocarcinoma.

## Additional files

**Additional file 1: Table S1.** Baseline characteristics of patients with lung adenocarcinoma in the validation cohort. Continuous data (age) was presented as mean ± standard deviation and categorical data as number (proportion of that the subgroup accounted for the whole group).

**Additional file 2: Table S2.** Cut-off value for immune cell fractions in the training cohort.

**Additional file 3.** Additional information.

**Additional file 4: Figure S1.** Survival analysis of the progression-free time stratified by immune cell infiltrating score in the training cohort (*P* < 0.001).

**Additional file 5: Table S3.** Results of univariate and multivariate Cox regression of overall survival in the validation cohort. HR = hazard ratio; CI = confidence interval.

**Additional file 6: Figure S2.** A: Survival analysis for patients (stage II-IV) who received chemotherapy stratified by immune cell infiltrating score in the training cohort (*P* = 0.004). B: Survival analysis for patients (stage II–IV) who did not receive chemotherapy stratified by immune cell infiltrating score in the training cohort (*P* = 0.002). C: Survival analysis for patients (stage II–IV) who received chemotherapy stratified by immune cell infiltrating score in the validation cohort (*P* = 0.084). D: Survival analysis for patients (stage II–IV) who did not receive chemotherapy stratified by immune cell infiltrating score in the validation cohort (*P* = 0.761).

**Additional file 7: Table S4.** Correlation between the immune infiltrating score and the expression of immune checkpoint regulators or inflammatory mediators in the validation cohort.

## Data Availability

All datasets were adopted in this study are available in the GEO (https://www.ncbi.nlm.nih.gov/geo/) and TCGA (https://portal.gdc.cancer.gov/) database.
