# Supplementary material for: Identification and validation of an immune cell infiltrating score predicting survival in patients with lung adenocarcinoma
Source: J Transl Med. 2019 Jul 8;17:217. doi: 10.1186/s12967-019-1964-6 (PMC6615164; doi:10.1186/s12967-019-1964-6)
Supplement: Supplementary file 1 — Additional file 1: Table S1. Baseline characteristics of patients with lung adenocarcinoma in the validation cohort. Continuous data (age) was presented as mean ± standard deviation and categorical data as number (proportion of that the subgroup accounted for the whole group). [file 12967_2019_1964_MOESM1_ESM.docx]

| Variable | Baseline characteristics (n=418) |
| --- | --- |
| Age | 65.8 ± 9.7 |
| Sex |  |
| Male | 187 (44.7) |
| Female | 231 (55.3) |
| Tumor stage |  |
| Stage I | 226 (54.1) |
| Stage II-III | 174 (41.6) |
| Stage IV | 18 (4.3) |
| Smoking Status |  |
| Current smoker | 35 (8.4) |
| Reformed smoker (<= 15 years) | 55 (13.2) |
| Reformed smoker (> 15 years) | 52 (12.4) |
| Non-smoker | 21 (5.0) |
| Unknown | 255 (61.0) |
| Adjuvant Chemotherapy |  |
| Yes | 146 (34.9) |
| No/unknown | 272 (65.1) |
| Histological Subtype |  |
| Micropapillary / Solid | 72 (17.2) |
| Acinar / Papillary | 105 (25.1) |
| Lepidic | 9 (2.1) |
| Others and unknown | 232 (55.6) |

Table S1: Baseline characteristics of patients with lung adenocarcinoma in the validation cohort. Continuous data (age) was presented as mean ± stand‎ard devia‎tion and categorical data as number (proportion of that the subgroup accounted for the whole group).
