# Supplementary material for: Identification and validation of an immune cell infiltrating score predicting survival in patients with lung adenocarcinoma
Source: J Transl Med. 2019 Jul 8;17:217. doi: 10.1186/s12967-019-1964-6 (PMC6615164; doi:10.1186/s12967-019-1964-6)
Supplement: Supplementary file 2 — Additional file 2: Table S2. Cut-off value for immune cell fractions in the training cohort. [file 12967_2019_1964_MOESM2_ESM.docx]

**Table S2:Cut-off value for immune cell fractions in the training cohort.**

| **Immune Cell type** | **Cut-off value** |
| --- | --- |
| B cells naive | 0.034747083651722 |
| B cells memory | 0.022850904986781 |
| Plasma cells | 0.24350404813495 |
| T cells CD8 | 0.20172887701428 |
| T cells CD4^+^ naive | 0.0075085703426141 |
| CD4^+^T cells memory resting | 0.051335094610022 |
| CD4^+^ T cells memory activated activated | 0.032968606537251 |
| T cells follicular helper | 0.0297630987179 |
| T cells regulatory | 0.017962377665642 |
| γδT cells | 0.024146444889069 |
| NK cells resting | 0.034033415083448 |
| NK cells activated | 0.020063808424143 |
| Monocytes | 0.026318677938711 |
| Macrophages M0 | 0.20327888818185 |
| Macrophages M1 | 0.057313585452216 |
| Macrophages M2 | 0.17023111146085 |
| Dendritic cells resting | 0.056905466127555 |
| Dendritic cells activated | 0.046908236520636 |
| Mast cells resting | 0.029099282767015 |
| Mast cells activated | 0.070255708375697 |
| Eosinophils | 0 |
| Neutrophils | 0.028640010668139 |
