# Supplementary material for: Identification and validation of an immune cell infiltrating score predicting survival in patients with lung adenocarcinoma
Source: J Transl Med. 2019 Jul 8;17:217. doi: 10.1186/s12967-019-1964-6 (PMC6615164; doi:10.1186/s12967-019-1964-6)
Supplement: Supplementary file 3 — Additional file 3. Additional information. [file 12967_2019_1964_MOESM3_ESM.docx]

**Additional information: The formula for the immune cell infiltrating score derived from the least absolute shrinkage and selection operator (LASSO) Cox regression in the training cohort. The immune cell fraction level divided by the cut-off value was valued as 0 or 1 in this immune infiltrating score formula.**

Plasma cells × (-0.224280801) + T cells CD8 × (0.009835404) + T cells CD4 memory resting × (-0.179926943) + T cells CD4 memory activated × (0.070710393) + T cells follicular helper × (0.136284645) + NK cells activated × (0.050648664) + Macrophage M0 × (0.307680005) + Macrophage M1 × (0.068630071) + Macrophage M2 × (0.129725939) + Dendritic cells activated × (0.218854217) + Mast cells resting × (-0.250907406) + Mast cells activated × (0.160597523) + Neutrophil × (0.187597462)
