# Supplementary figures and images for: Identification and validation of an immune cell infiltrating score predicting survival in patients with lung adenocarcinoma
Source: J Transl Med. 2019 Jul 8;17:217. doi: 10.1186/s12967-019-1964-6 (PMC6615164; doi:10.1186/s12967-019-1964-6)

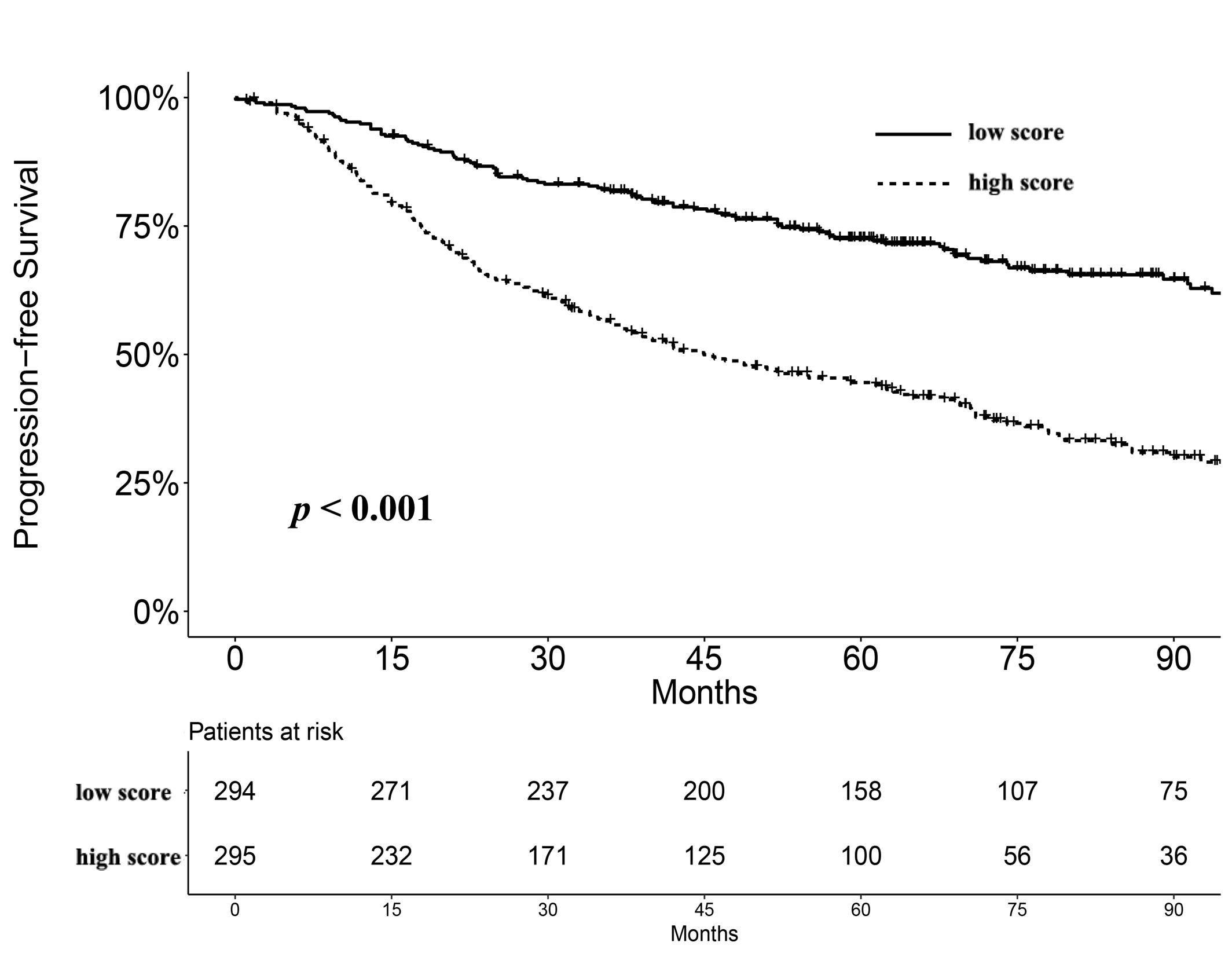

Supplement: Supplementary file 4 — Additional file 4: Figure S1. Survival analysis of the progression-free time stratified by immune cell infiltrating score in the training cohort (P < 0.001). [file 12967_2019_1964_MOESM4_ESM.tif]

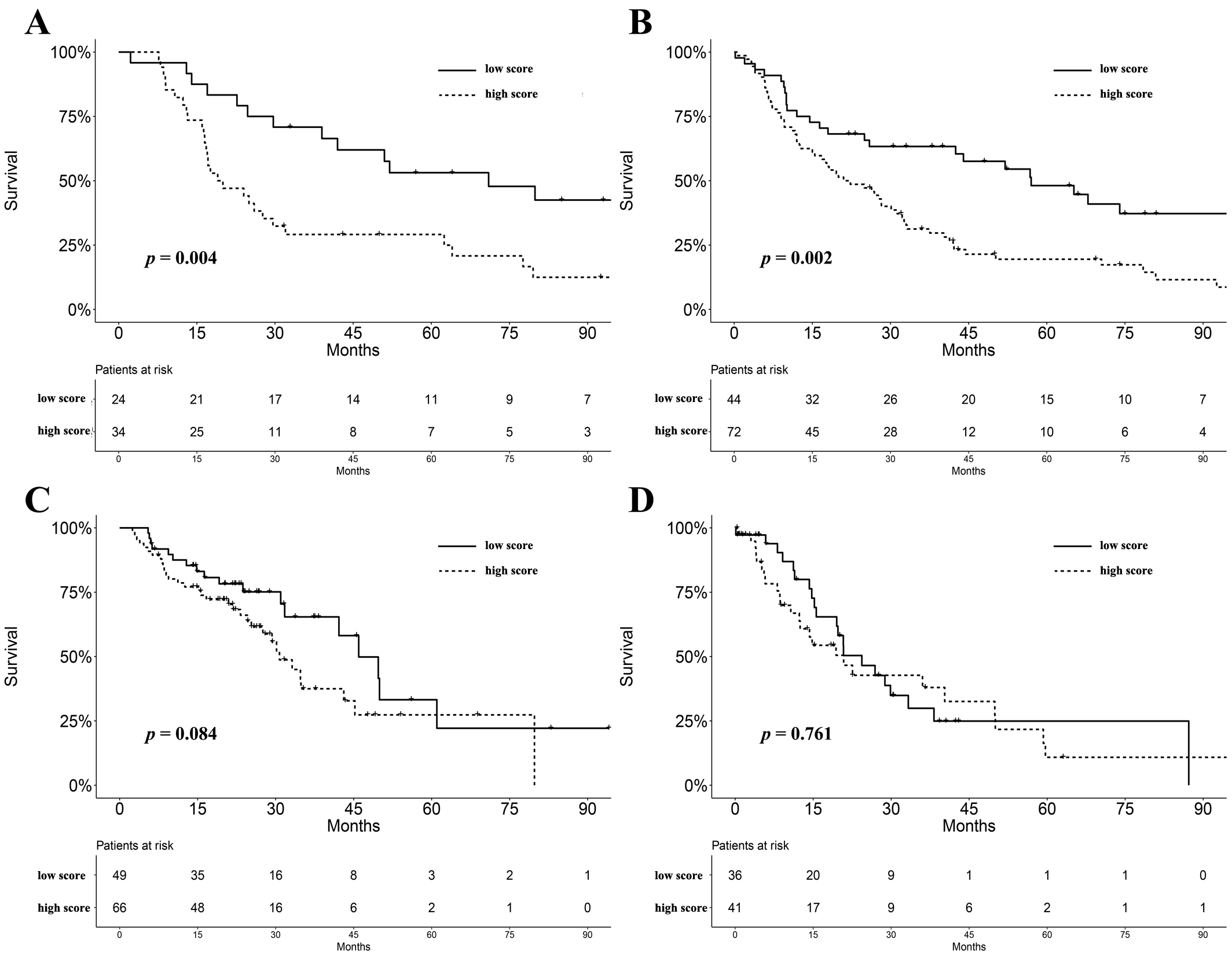

Supplement: Supplementary file 6 — Additional file 6: Figure S2. A: Survival analysis for patients (stage II-IV) who received chemotherapy stratified by immune cell infiltrating score in the training cohort (P = 0.004). B: Survival analysis for patients (stage II–IV) who did not receive chemotherapy stratified by immune cell infiltrating score in the training cohort (P = 0.002). C: Survival analysis for patients (stage II–IV) who received chemotherapy stratified by immune cell infiltrating score in the validation cohort (P = 0.084). D: Survival analysis for patients (stage II–IV) who did not receive chemotherapy stratified by immune cell infiltrating score in the validation cohort (P = 0.761). [file 12967_2019_1964_MOESM6_ESM.tif]
