# Supplementary material for: Identification and validation of an immune cell infiltrating score predicting survival in patients with lung adenocarcinoma
Source: J Transl Med. 2019 Jul 8;17:217. doi: 10.1186/s12967-019-1964-6 (PMC6615164; doi:10.1186/s12967-019-1964-6)
Supplement: Supplementary file 5 — Additional file 5: Table S3. Results of univariate and multivariate Cox regression of overall survival in the validation cohort. HR = hazard ratio; CI = confidence interval. [file 12967_2019_1964_MOESM5_ESM.docx]

Table S3: Results of univariate and multivariate Cox regression of overall survival in the validation cohort. HR = hazard ratio; CI = confidence interval.

| Cohort | Validation cohort | | | | | |
| --- | --- | --- | --- | --- | --- | --- |
| Variable | Univariate | | | Multivariate | | |
|  | HR | 95% CI | *P*-value | HR | 95% CI | *P*-value |
| Immune infiltrating score | 2.900 | 1.536-5.475 | 0.001 | 2.812 | 1.443-5.478 | 0.002 |
| Age | 1.007 | 0.990-1.024 | 0.417 | 1.016 | 0.999-1.033 | 0.063 |
| Sex |  |  | 0.272 |  |  | 0.615 |
| Female | Reference |  |  | Reference |  |  |
| Male | 1.199 | 0.867-1.657 | 0.272 | 1.088 | 1.873-7.217 | 0.615 |
| TNM stage |  |  | <0.001 |  |  | <0.001 |
| Stage I | Reference |  |  | Reference |  |  |
| Stage II-III | 2.985 | 2.105-4.233 | <0.001 | 2.905 | 2.047-4.124 | <0.001 |
| Stage IV | 3.756 | 1.942-7.264 | <0.001 | 3.677 | 1.873-7.217 | <0.001 |
| Smoking history |  |  | 0.936 |  |  |  |
| Yes | Reference |  |  |  |  |  |
| No | 1.107 | 0.575-2.135 | 0.761 |  |  |  |
| Unknown | 0.984 | 0.697-1.390 | 0.928 |  |  |  |
| Histological Subtype |  |  | 0.069 |  |  |  |
| Micropapillary / Solid | Reference |  |  |  |  |  |
| Acinar / Papillary | 0.784 | 0.522-1.178 | 0.241 |  |  |  |
| Lepidic | 0.333 | 0.080-1.391 | 0.132 |  |  |  |
| Others and unknown | 0.551 | 0.337-0.900 | 0.017 |  |  |  |
| EGFR mutation |  |  | 0.672 |  |  |  |
| No | Reference |  |  |  |  |  |
| Yes | 1.327 | 0.712-2.473 | 0.373 |  |  |  |
| Unknown | 1.046 | 0.744-1.470 | 0.798 |  |  |  |
| Tumor Location |  |  | 0.771 |  |  |  |
| Left | Reference |  |  |  |  |  |
| Right | 1.124 | 0.804-1.570 | 0.494 |  |  |  |
| Unknown | 0.940 | 0.294-3.003 | 0.917 |  |  |  |
