# Supplementary material for: Identification and validation of an immune cell infiltrating score predicting survival in patients with lung adenocarcinoma
Source: J Transl Med. 2019 Jul 8;17:217. doi: 10.1186/s12967-019-1964-6 (PMC6615164; doi:10.1186/s12967-019-1964-6)
Supplement: Supplementary file 7 — Additional file 7: Table S4. Correlation between the immune infiltrating score and the expression of immune checkpoint regulators or inflammatory mediators in the validation cohort. [file 12967_2019_1964_MOESM7_ESM.docx]

|  | Validation Cohort | |
| --- | --- | --- |
| Variable | *P*-value | Pearson correlation coefficient |
| CD47 | 0.002 | 0.154 |
| PD-1 | 0.716 | 0.018 |
| CLTA4 | 0.820 | -0.011 |
| LAG3 | 0.017 | 0.117 |
| IDO1 | 0.003 | 0.146 |
| TIM-3 | 0.301 | 0.051 |
| IFNA1 | 0.419 | 0.040 |
| IFNA2 | 0.140 | 0.072 |
| IFNB1 | 0.002 | 0.149 |
| IL-1A | 0.005 | 0.136 |
| TNFA | 0.010 | 0.126 |
| IL-1B | <0.001 | 0.261 |
| IL-6 | 0.007 | 0.131 |
| IL-10 | 0.534 | 0.031 |
| IL-12A | 0.137 | 0.073 |
| IL-12B | 0.411 | -0.040 |

Table S4: Correlation between the immune infiltrating score and the expression of immune checkpoint regulators or inflammatory mediators in the validation cohort.
